# Study on intervention effect of Wearable Cyborg HAL through narrative analysis

**DOI:** 10.3389/fpsyg.2025.1462072

**Published:** 2025-04-02

**Authors:** Shiori Ikemoto, Yoshiyuki Sankai, Takeru Sakurai

**Affiliations:** ^1^Research and Development Department, CYBERDYNE Inc., Tsukuba, Japan; ^2^Center for Cybernics Research, University of Tsukuba, Tsukuba, Japan; ^3^Institute of Systems and Information Engineering, University of Tsukuba, Tsukuba, Japan

**Keywords:** Wearable Cyborg HAL, Cybernics Treatment, Techno Peer Support, Neuro HALFIT, patient reported outcome, narrative analysis, mutual feedback structure model in physical, mental, and social aspects

## Abstract

**Background:**

The medical effectiveness of Cybernics Treatment with the Wearable Cyborg HAL (Hybrid Assistive Limb) has been verified, and its treatment method has started to be used in many countries around the world. However, the focus of medical evaluations has predominantly been on simple measurement evaluation of physical function, while Patient Reported Outcome (PRO), which encompass various evaluation axes of patients, remain largely unexplored. The mental/psychological field has the potential to develop cutting-edge fields targeting individuals. As the social implementation of HAL progresses, it is important to capture how the physical intervention by HAL affects not only the physical function but also the mental state and social activities of individual users. This approach will help us understand the various aspects of the effects of Cybernics Treatment using HAL.

**Objective:**

In order to elicit deeper narratives of HAL users, this study aims to capture how HAL users with limited physical functionality have changed physically, mentally and socially due to using HAL, through a narrative analysis utilizing counseling methods. Based on the results, the significance of using HAL will also be discussed.

**Methods:**

We analyzed the narratives of nine HAL users who received the services of “Neuro HALFIT.” During the interview survey, we also visualized the narratives using mathematical engineering methods (cluster analysis, dendrogram) based on the similarity distance matrix between the association items and elicited them by deepening the narratives through counseling methods and captured the state of change in the physical, mental, and social aspects of the subjects.

**Results:**

The results suggested that “Neuro HALFIT” improved physical function and provided mental and social improvements. These three aspects influenced and circulated each other, advancing toward improvement and enhancement, and the “Mutual feedback structure model in physical, mental, and social aspects of patients” was proposed and presented. Based on the above analysis, it was considered that the greatest significance of using HAL was to help many people with fixed disabilities or those who were considered to have no treatment to turn the process of this model without losing hope and to participate in society with a sense of fulfillment in their lives.

## 1 Introduction

**Figure 1:**
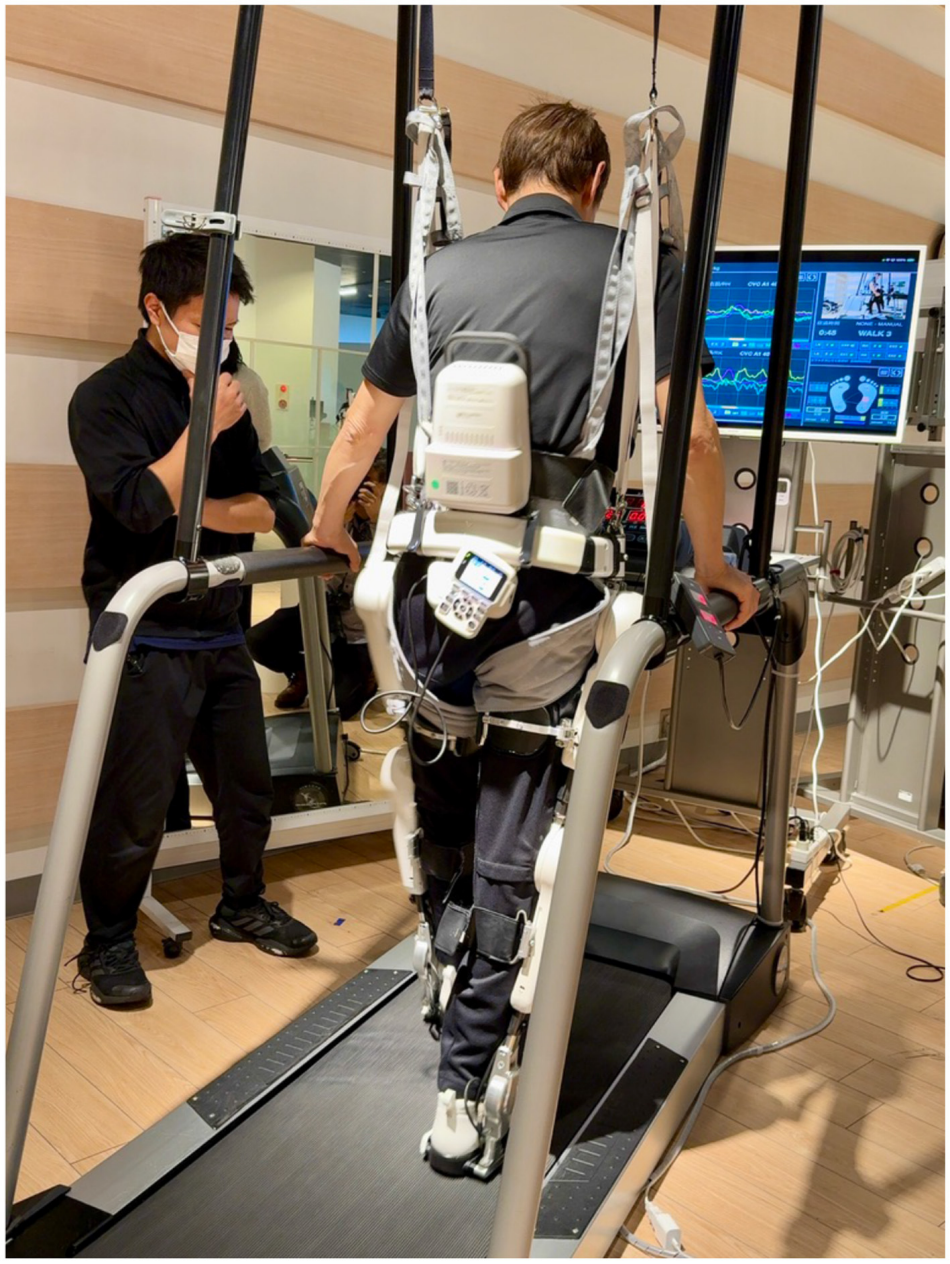
Wearable Cyborg HAL.

Cybernics Treatment with Wearable Cyborg HAL (Figure 1) was created as a new treatment method for patients with diseases of the body and nervous system (intractable neuromuscular diseases for which no treatment had previously been available; Sankai and Sakurai, 2018; Nakajima et al., 2021), and after clinical evaluation and clinical trials, HAL is now being used in 20 countries around the world. In Germany, Cybernics Treatment was the world's first treatment for spinal cord injury to be covered by public workers' compensation insurance (2013), and in Japan, it has been covered by public medical insurance for 8 progressive intractable neuromuscular diseases (2016) after randomized controlled crossover trial (NCY-3001 trial; Nakajima et al., 2021) and 10 diseases (2023) with the addition of 2 more diseases, including intractable spastic paraplegia (CYBERDYNE Inc., 2025).

When evaluating the efficacy of the HAL treatments, physical function is currently the primary endpoint (clinical trials for neuromuscular diseases), which is a traditional endpoint. Distance for 2 min (or 6 min), 10 m walking speed, etc. are measured and statistically analyzed. In this way, the statistical properties of the effect of a treatment on a patient population can be clarified. However, individual patients have diverse backgrounds, and there is a limit to capturing diverse patient information from the perspective of medical statistics for a group. In recent years, in addition to conventional statistical and average medicine for populations, precision medicine that takes genetic and other characteristics into account has emerged, and it is necessary to connect this to individualized medicine and health from the diverse perspectives of individual patients in the future. The mental/psychological field including mental health has the potential to develop as a cutting-edge pioneering area for the individual. In recent years, there has been an increasing emphasis on the important role of patient motivation in determining outcomes in traditional rehabilitation and new medical technologies (Maclean et al., 2000; Kitajima et al., 2014a), as well as the perspective of looking at single person from multiple perspectives (Kitajima et al., 2014b). In particular, Patient Reported Outcome (PRO) studies on the patient side have become increasingly important in recent years, and the U.S. FDA issued guidance in 2009 indicating PRO measurement (US Department of Health and Human Services Food and Drug Administration, 2009).

Functional improvement therapy with HAL, which has emerged as one of the most advanced scientific technologies mentioned above, focuses on improving physical function (walking function). Through the efforts to promote functional improvement with HAL to date, interestingly, we have noticed a mental change in the users. As social implementation of HAL progresses worldwide, it is very important to understand how interventions on the user/patient's physical body affect their mental health on an individual level, and furthermore, social aspects should also be taken into consideration.

Therefore, we have begun the challenge of proposing a method and applying it to actual situations to capture the possibility that physical interventions using HAL for individual users will improve their “physical functions,” which in turn will lead to improvements in their “mental health,” and then to improvements in their “social activities” (Ikemoto et al., 2022). This is different from the conventional evaluation based on simple walking distance and time alone to capture changes in physical condition as patient information.

There is an increasing number of reports on physical changes in HAL users from the viewpoint of providers of treatment programs. However, very few PRO studies have focused on the psychological aspects of HAL users. One study used FAC (Functional Ambulatory Categories) as the primary endpoint, with PRO evaluation SF-8 (Health-related Quality of Life) and POMS (Profile of Mood States) as secondary endpoints (Watanabe et al., 2016). Another study has investigated and analyzed the PROs of HAL users quantitatively and qualitatively (Ikemoto et al., 2022). But those are very few in number. We believe that clarifying how HAL users experience HAL and how they feel its effects will have important significance from the perspective of disseminating information toward the realization of a “Techno Peer Support” society where technology like HAL and people coexist and support each other (CYBERDYNE Inc., 2024).

Narrative analysis is a method of revealing the inner world of an individual generated through the subject's narratives. It is sometimes pointed out that narratives are only one case study because they are individual narratives, but when focusing on individual patients, it is possible to capture information that cannot be captured by medical statistical methods targeting groups. This information can be used to provide better treatment and mental support for patients with a variety of problems.

In this study, narrative analysis will be conducted by psychological professional utilizing counseling methods with the aid of PAC analysis (Analysis of Personal Attitude Construct; Naito, 2024) for HAL users. Narratives will be elicited by looking at the visualized dendrogram and the process of cluster cohesion will also be textualized, and the counseling method will be used to generate deeper narratives and devise a way to recall response shifts (Ito, 2008) due to awareness in real time. These features will be used to construct a method that allows psychological professionals and individual HAL users to share information that has been analyzed and organized to understand what is happening during the ongoing period of treatment intervention and daily life.

In addition, by investigating, analyzing, and visualizing the narratives of HAL users over the medium to long term from the viewpoint of time using the above approach, the purpose of this study is to clarify how HAL users with limited physical functions have physically changed, and what kind of mental and social changes have occurred as a result, as intervention effect of HAL, and to consider the significance of using HAL through these.

## 2 Methods

### 2.1 About the Wearable Cyborg HAL

Medical HAL is currently used in the medical field for “Cybernics Treatment” to improve the functions of the cranial nerve and muscle systems. By going through a program using HAL, an interactive BioFeedback (iBF) loop [brain → spinal cord → motor neurons/motor nerves → muscles → HAL] and [HAL → proprioceptors → sensory nerves → spinal cord → brain] is formed between the body and HAL (Standring, 2016). In other words, the musculoskeletal system is moved by HAL operating in synchrony with the wearer's motion intention. During this process, proprioceptors, including muscle spindles among others, are activated through muscle extension. In voluntary movements assisted by HAL, with almost no muscle load, the signal loop of the mechanism of action is continuously engaged, thereby promoting functional recovery and regeneration (Sankai and Sakurai, 2017; Saotome et al., 2022).

### 2.2 “Neuro HALFIT” and Robocare Center

“Neuro HALFIT” is a neuromuscular functional improvement program using non-medical HAL systems, including the lower limb type [dual-leg (Ezaki et al., 2021; Kadone et al., 2020), single-leg (Watanabe et al., 2020)], single-joint type (Shimizu et al., 2019), and lumbar type (Yasunaga et al., 2021; Kato et al., 2022). It is delivered by specialized staff, such as physical therapists, occupational therapists, and certified health exercise instructors, who customize the program to meet individual needs (ROBOCARE CENTER Group, Neuro HALFIT, 2024). This program serves individuals with cerebrovascular disorders, spinal cord injuries, and neuromuscular diseases, for which no fundamental treatment exists, as well as those seeking to prevent frailty. Each session lasts either 70 min (16,600 JPY) or 90 min (17,600 JPY). Users have the option to combine multiple HAL types within each session. As of July 2024, this service is available at 18 locations across Japan, including the Tsukuba Robocare Center, operated by CYBERDYNE (ROBOCARE CENTER Group, Robocare Center Locations and Fees, 2024). These Robocare Centers do not offer the standard rehabilitation services typically available in hospitals. Instead, they focus exclusively on HAL-based neuromuscular functional improvement programs tailored for individuals in the maintenance phase of rehabilitation.

### 2.3 Research participants

The authors, in consultation with HALFIT's specialized staff, identified potential research participants. Participants were selected from HAL users enrolled in the “Neuro HALFIT” program at the Tsukuba Robocare Center based on the following inclusion criteria. We decided to include those who were able to cooperate with and physically endure a lengthy interview survey, those who communicate well, and those who had been continuing the “Neuro HALFIT” program for several months or longer, thus ensuring to exclude those who had not yet experienced any changes because they had only been coming for a short period of time. The nine participants who were selected were diverse in age, gender, diagnosis, physical condition, length of time since the onset of their illness, and duration of HAL use. Consent was obtained from all nine participants, and all nine were included without exclusion to avoid bias. In psychological research where narrative interviews are conducted, nine participants are considered sufficient.

### 2.4 Survey period and timing

The survey period was from October to December 2015 (2 cases) and from September 2020 to January 2021 (7 cases). When participants came to the Tsukuba Robocare Center, we conducted the survey and asked them to cooperate for research in two sessions (Sessions 1 and 2).

### 2.5 Ethical considerations

Before the survey, we provided the participants with a document that stated: “The purpose and content of the survey would be used for research purposes, the conversation will be recorded, the participation is voluntary, no disadvantages would result from participation or non-participation, data would be anonymized, data would be stored for a certain period until it is disposed of, the survey could be discontinued even in the process if the participants felt burdened, and if the participants have difficulty writing or reading the researcher will provide necessary support.” Since ethical review is required at the site where the research is conducted, this study underwent research ethics review by CYBERDYNE Inc., which manages and operates the Tsukuba Robocare Center (however, the ethics review committee member of the University of Tsukuba, the joint research partner, serves as the ethics review committee chairman). This study conforms to the guidelines of the Japanese Psychological Association, was approved by CYBERDYNE's internal Research Ethics Committee (approval number: 150003), and is being conducted under the Declaration of Helsinki.

### 2.6 Survey method

The interviews were conducted by one of the authors, a clinical psychologist involved in the study. By carefully listening to each individual's narrative using counseling techniques, the researcher was able to deeply explore the individual's inner world. An interview survey was conducted with the aid of the PAC analysis (Analysis of Personal Attitude Construct) method (Naito, 2024). This method allows the researcher to obtain the overall vital variables and factors recalled from the informants' long-term memory and interpret the data phenomenologically through conversation to learn about an individual's inner world.

**Figure 2:**
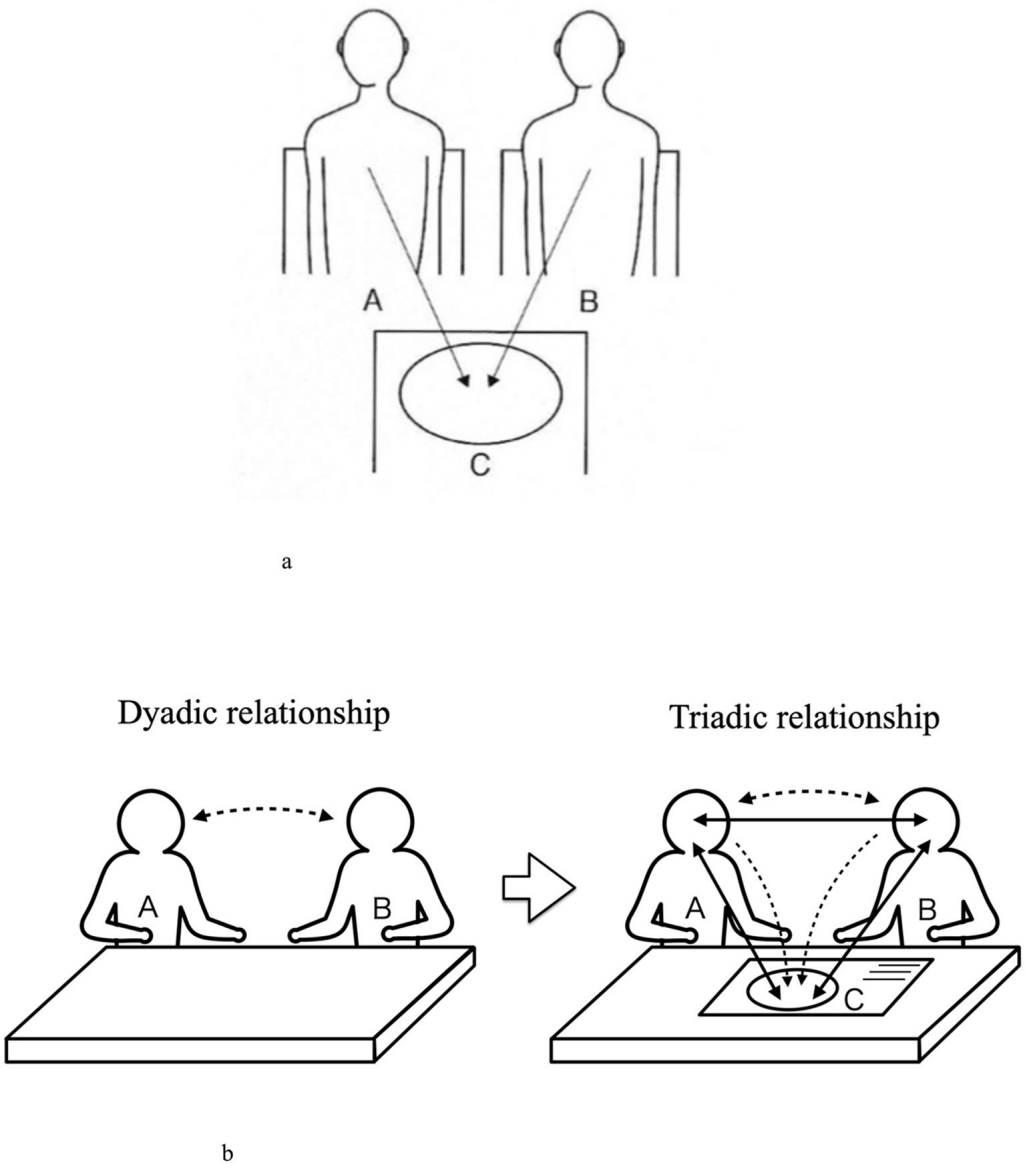
**(a)** Triadic relationships Model I. **(b)** Visual narrative that makes up Triadic relationship.

Narrative refers to “the act of speaking through words and what was spoken,” whereas visual narrative is conversing through visual images or visual images and words. While face-to-face dialogue could sometimes be confronting, in a visually mediated triadic relationship, two persons are next to each other and see the same thing. The latter is more likely to create a sympathetic and empathic atmosphere (Yamada, 2019). A triadic relationship is a relational theory emphasizing the mediating function [mediator or medium] that connects people. Yamada describes three models of Triadic relationships: Model I (communication in line relationships; Figure 2a), Model II (communication in matchmaker relationships), and Model III (communication in watchful waiting relationships; Yamada, 2021). This paper shows a “Visual narrative that makes up the Triadic relationship” (Figure 2b) to assist in understanding.

Since seven of the nine research participants in this study had difficulty writing due to disabilities related to their diseases and requested that the researcher write on their behalf, the participant and the researcher were positioned side by side. The same position was also chosen for the two who did not request to write instead of them. This corresponds to the “Triadic Relationship Model I,” which co-generates the narratives.

This study was conducted with the aid of the PAC analysis method (Naito, 2024). The researcher proceeded with the interview in a counseling approach, such as sympathizing with the narratives told by the participants. At times, the researcher promoted the narrative or asked questions to deepen the conversation, but the researcher ensured that such responses aligned with the flow of the narrative. The study took an original approach by conducting the interviews in a counseling style while taking the Triadic Relationship Model I position.

The interview questions were: “What physical and mental changes have you experienced through using HAL? Did those changes cause any difference in your behaviors or lifestyle?”. The phrase “social changes” is difficult to understand, so the researcher replaced it with “behavioral and lifestyle changes.” During the interview survey, the participants kept a printed copy of the question on hand so they could check it at any time.

### 2.7 Procedure

The following (1) to (6) are tasks to be performed proactively by the participants. The researcher will guide them through the process, but will not guide them in the content of their responses.

**<Interview Survey: Session 1>**

1) Writing out association items: After hearing the question, the participant wrote down any answers that came into his/her mind one by one on a card as “association items.” This process continued until the participant no longer found something to write about the question. If the participant had difficulty writing, the researcher provided assistance by transcribing participants' responses on his/her behalf.
2) Sort association items by importance: The participant looked at all the association items written down and arranged the cards in order from the most important (smaller number) to the least important (larger number).
3) Similarity ratings between association items and creation of similarity distance matrices: The participant was asked to judge how similar or different association items are from other association items based on their intuitive images. The researcher asked participants to answer this on a 7-point scale: very close = 1, reasonably close = 2, somewhat close = 3, neither close nor far = 4, somewhat far = 5, relatively far = 6, and very far = 7. The researcher then created a similarity distance matrix table based on those numbers.
4) Generating a dendrogram by cluster analysis: The researcher performed a cluster analysis of the similarity distance matrix using the Ward method with statistical software (SPSS Ver25) and generated a dendrogram (tree diagram).

**<Interview Survey: Session 2>**

5) Interviews by clusters (collection of narratives) and naming of clusters: The researcher, together with the participant, examines the calculated dendrogram, a tree diagram that visually represents the relationships between association items based on their similarity distances. In this structure, association items that are close in distance are grouped into the same cluster, clusters that are close to each other are merged into larger clusters, and ultimately, all items are consolidated into a single large cluster. While reviewing the dendrogram, the researcher interviews the participant cluster by cluster regarding the content of the association items. To elicit deep narratives, the researcher responds using counseling techniques, actively engaging in the conversation to encourage reflection and insight. The participant conceptualizes the meaning of each cluster and assigns a cluster name accordingly. As smaller clusters merge into larger clusters, the participant also names these newly formed larger clusters. This iterative process continues until all clusters are fully integrated into a single, overarching cluster. After the clustering process is complete, the researcher conducts supplementary questioning to further explore and collect even deeper narratives.
6) Images of association items: For each association item, the researcher asked the participant whether it sparks positive (+) images, negative (–) images, or somewhere in between (+/–), and the researcher or the participant wrote down those evaluations on each card.

**<Converting narratives into texts>**

7) Converting narratives into texts by the researcher: The researcher converted the participant's narratives obtained from the interview into text, following the order of the narratives. In this step, the researcher also recorded the process of turning each item into clusters and deepening the narratives. The researcher then turned both processes into texts.

**<Grouping of all association items>**

8) Grouping of all association items by the researcher: All association items obtained from the participant were grouped by the researcher into three perspectives: physical, mental and social. A list of association items and a classification table for each of the three perspectives were created.

## 3 Results

### 3.1 Profile of participants

The participants were nine HAL users visiting Tsukuba Robocare Center. “Age,” “Gender,” “Diagnosis,” “FAC (Functional Ambulation Categories),” “WISCI II (Walking index for spinal cord injury II),” “Period after onset,” and “HAL usage period” are shown in Table 1.

**Table 1:** Research participants profiles.

**Participants**	**Age**	**Gendar**	**Diagnosis (Supplementary)**	**FAC**	**WISCI II**	**Period after onset**	**HAL usage period**
A	30s	M	Head Injury (walker use)	4	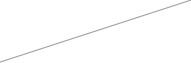	16 years	5 years
B	60s	F	Stroke/ICH	5	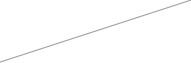	4 years	4 years
C	60s	F	Polio (Both Lower limb dysfunction)	4	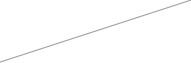	58 years	5 years
D	60s	M	Stroke/CI	5	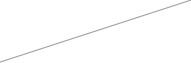	2 years	2 years
E	70s	M	Stroke/ICH	5	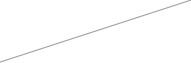	7 years	5 years
F	30s	M	SCI/C4-C6 (wheelchair use)	0	0	11 years	10 years
G	30s	F	Charcot-Marie-Tooth disease	5	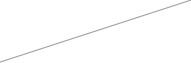	27 years	3 years
H	60s	M	SCI/L1-L2 (wheelchair use)	3	16	1 year 5 months	8 months
I	40s	M	SCI/C4-C6	5	20	1 year 9 months	1 year 3 months

ICH, intracranial hemorrhage; CI, cerebral infarction; SCI, spinal cord injury; FAC, Functional Ambulation Categories. Used as an evaluation index to determine the degree of independence of walking ability easily in clinical settings (classified into 6 levels from 0 to 5: 0 = Non-functional ambulator, 1 = Ambulator, dependent on physical assistance—level I, 2 = Ambulator, dependent on physical assistance—level II, 3 = Ambulator, dependent on supervision, 4 = Ambulator, independent level surface only, 5 = Ambulator, independent); WISCI II, Walking index for spinal cord injury II. Walking index for spinal cord injury patients (21 levels from 0 to 20, where 0 = unable to walk to 20 = no need for any aid, brace, or assistance).

### 3.2 Case study: narrative analysis of participant G

#### 3.2.1 On describing the case study of participant G

This study used counseling approaches and mathematical engineering methods for narrative analysis that involves a large number of descriptions, and in the interest of brevity, we decided to describe only one case in detail to illustrate the analysis procedure that was implemented. As described in “Section 1”, a randomized controlled crossover trial that included eight diseases: spinal muscular atrophy, bulbospinal muscular atrophy, amyotrophic lateral sclerosis, Charcot-Marie-Tooth disease, distal myopathy, inclusion body myositis, congenital myopathy, and muscular dystrophy (Nakajima et al., 2021) was conducted to demonstrate the effects of HAL on the physical function of its users. Since participant G had Charcot-Marie-Tooth disease and was the only participant with a neuromuscular disease included in the HAL NCY-3001 RCT, we thought it would be meaningful to report in detail how she was changing with the use of the HAL, in terms of research from a different perspective than the RCT. As such, we describe the details of G's case study.

#### 3.2.2 Profile of G

Participant G is a female participant in her 30s. When she was seven, a doctor gave her a final diagnosis of Charcot-Marie-Tooth disease. Her physical disability level in her certificate is grade three in the upper limb and grade five in the lower limb. Twenty-seven years have passed since she first realized her symptoms, and she has been using HAL for 3 years. She is capable of walking without a cane, but 4 years ago, she felt the disease was progressing, as she found it challenging to continue her work that requires tasks in a standing position. When thinking about quitting her job, she learned about HAL and visited the Robocare Center for the first time. She conducts gait training with HAL Lower Limb Type, arm, hand, and ankle movement training using HAL Single Joint Type to continue her work. She visits the center once every 1 to 2 weeks.

#### 3.2.3 Flow of the interview survey for G

Participant G came up with 27 association items. In the interview conducted while the researcher and Participant G were looking together at the dendrogram generated through cluster analysis, the researcher asked Participant G to talk about the contents of each cluster. The researcher then requested that Participant G consider the similarities of items in the same cluster and write a name that suited that cluster. The researcher numbered those clusters from the top to bottom by CL1-CL7. The researcher carefully listened to the narratives and asked questions as appropriate. The_researcher and Participant G repeated the process as the researcher connected the clusters with Participant G. After naming a word to summarize the one cluster that the_researcher and Participant G formed at the end, the researcher asked what Participant G thought about the interview survey.

#### 3.2.4 G's narrative results

**Figure 3:**
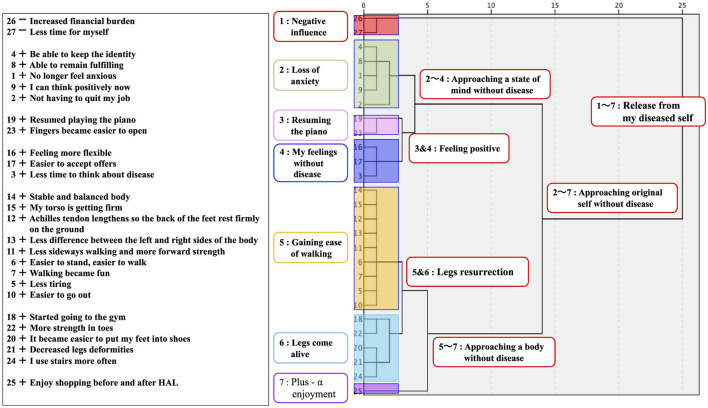
Participant G's dendrogram.

Figure 3, “Participant G's Dendrogram,” is composed of the “association items,” “order of importance (1–27),” and “image of association items (+/–/±)” overlapped on a dendrogram that was generated. The researcher started the interview sequentially from Cluster 1 to Cluster 7. The narratives generated while linking each cluster are shown in Table 2, “Participant G's Narrative results.” The questions that the researcher asked research participant to elicit deep narratives in a counseling manner are shown in < > in Table 2. These questions show how they were able to collect deep narratives.

**Table 2:** Participant G's narrative results.

**Cluster labeling/importance ranking/images/association items**	**Narrative (Researcher's utterances are noted with < >)**
[CL1: Negative influence] 26-Increased financial burden 27-Less time for myself	The lower the HAL fee, the better, but it's not so much a financial drain. I come from far away and sometimes I must lose a day off during the week, so I have less time for myself. <Can you tell me how much of a burden that is for you?> It's such a small negative impact that it's drowned out compared to the benefits. I get paid enough to come here, but some people have difficulty with time and money. I wish HAL could be done more readily available at a nearby hospital.
[CL2: Loss of anxiety] 4 + Be able to keep the identity 8 + Able to remain fulfilling 1 + No longer feel anxious 9 + I can think positively now 2 + Not having to quit my job	Of all the changes, the biggest was that I no longer feel anxious. I had been thinking that I would have to quit my job because my body was getting so tight, but before I knew it, that worry had disappeared. I didn't have to quit my job. <Can you tell me a little more about your job?> I stand almost a day at work. I guess I'm used to it, it's always moving so I don't get tired. I'm not married and live alone, so my job is the foundation of my life. It was a reason to live and one of my identities, and I could keep it that way. I don't think I have to give up the sense of fulfilling anymore. <Important words like “reason to live, identity and fulfilling” have come up, but other than that, has there been any change in your feelings?> It was hard for me to go out when I had difficulty moving, but I have become somewhat more outgoing, and I am also able to think more positively.
[CL3: Resuming the piano] 19 + Resumed playing the piano 23 + Fingers became easier to open	Training my hands and arms with HAL Single joint has made it a little easier to open my fingers. I couldn't play the piano anymore, so I gave up and didn't even touch the piano for a long time, but recently I had a change of heart and I wondered if I didn't have to play the piano like everyone else, so I bought a piano. It's a cheap electronic one. I can't play with both hands or play chords at all, but I bought some scores for beginners, and I play it now and then. <How do you feel about playing the piano again?> I thought I liked the piano after all, and I thought it was so much fun to follow notes with my eyes in a book or something and then move my body to make the sound. <That's good. Can you tell me a little more about when you quit playing the piano?> The thing that stuck with me the most was that I had to stop playing the piano, which I had started when I was in kindergarten. When I was in elementary school, the shape of my hands gradually became deformed. I think the first thing I gave up in my life was the piano. So, when I got better and asked what I wanted to do, it was definitely to play the piano.
[CL4: My feelings without disease] 16 + Feeling more flexible 17 + Easier to accept offers 3 + Less time to think about disease	I don't think about disease anymore. It's not zero, but I have less time to think. As an adult, I kept thinking about it, especially as an adolescent in middle school and high school. I've been thinking about it for a long time, like... maybe I didn't even listen to it in class. I even wondered how I was able to get credits for it. It's something I can't solve even if I think about it, but I guess it was a vague anxiety. <You've been thinking that way. What kind of changes have you seen in your mindset as you spend less time thinking about your disease?> When your fever goes up and you hit 37.5, it's hard to think about “Let's do that tomorrow, let's do this” But when your fever goes down and you hit 37.5, you think, “What am I going to do tomorrow?” The same 37.5 may be a sensory problem, but I think the feeling is different between 37.5 when it gets worse and 37.5 when it gets better. My disease is progressive, so there must be some part of it that gets worse, but now that I'm clearly getting better with HAL, I feel that my fever has gone down a bit from its peak. As I mentioned in the association item, I think my feelings have become more flexible. It may be due to my age, but I don't mind if things aren't perfect. It has become easier to accept invitations from friends.
[CL5: Gaining ease of walking] 14 + Stable and balanced body 15 + My torso is getting firm 12 + Achilles tendon lengthens so the back of the feet rest firmly on the ground 13 + Less difference between the left and right sides of the body 11 + Less sideways walking and more forward strength 6 + Easier to stand, easier to walk 7 + Walking became fun 5 + Less tiring 10 + Easier to go out	I usually drive a regular car (not a modified car), but now that my legs are in better condition, I feel like I should walk a little instead of driving. I enjoy walking and feel the change in walking. Before, I had a lot of lateral sway in my walk, I couldn't move forward, and my knees would hurt. I couldn't walk efficiently, so I thought it might be better to walk backwards in this situation, so I tried it once (laughs). The side-to-side swaying became less and the forward force was greater. I feel like my energy is being used to move forward. My torso is getting stronger and more balanced, which makes me less tired.
[CL6: Legs come alive] 18 + Started going to the gym 22 + More strength in toes 20 + It became easier to put my feet into shoes 21 + Decreased legs deformities 24 + I use stairs more often	My legs were deformed and there was a difference between the right and left sides, which made my shoes difficult to put on. The deformity has come off a bit and the shoes are more comfortable. It became easier to move and act, so I started going to the gym. I go once a week. I don't like being crowded, so I go first thing in the morning on my days off. <Have you been acting so actively before?> I think I have an active personality by nature, but if I wasn't ill, I would be too active and think I might have died in an accident somewhere (laughs). Rather, I think the disease has restricted it well, making it just right. In addition, I often use the stairs. I live in an apartment, and I take the stairs to the 4th floor. I thought it was exercise and started doing so. Not every time, but I use it more often.
[CL7: Plus—α enjoyment] 25 + Enjoy shopping before and after HAL	I enjoy shopping before and after coming to HAL. I wonder what I should have for lunch. This shopping center has everything, so it's very convenient.
[CL3 and 4] Feeling positive	What CL3 “Resuming the piano” and CL4 “My feelings without disease” have in common is that they are “Feeling positive”? I started playing the piano again because I felt positive and wanted to try something.
[CL5 and 6] Legs resurrection	I think the CL5 “Gaining ease of walking” and CL6 “Legs come alive” can be summarized as “Legs resurrection.”
[CL2 and 3 and 4] Approaching a state of mind without disease	CL3 and 4 “Feeling positive” and CL2 “Loss of anxiety” are just like a set. <Like a set?> Being less anxious and more positive is like spending less time thinking about the disease and feeling more positive. The feeling is that I have returned to the carefree mental state of my childhood before I got the disease, so I would like to sum it up as “Approaching a state of mind without disease.”
[CL5 and 6 and 7] Approaching a body without disease	CL5 and 6 “Revival of the legs” and CL7 “Plus—α enjoyment” refer to the fact that my legs feel better and I actively enjoy shopping when I come here, so I would like to summarize them as “Approaching a body without disease.”
[CL2-7] Getting closer to my original disease-free self	CL2 and 3 and 4 “Approaching a state of mind without disease” and CL5 and 6 and 7 “Approaching a body without disease” remind me of my original self, pre-disease state of mind and body as healthy as I was born. <Is it your original self?> I had a sense that there were two parts of me: original self, and self who has become diseased and restricted and withdrawn, and there was always a conflict between them. It's getting better now, and I feel like I'm getting closer to my disease-free body, or I'm in a state of mind where I don't have to think about the disease, and I'm in the process of getting my original self back, so I guess I'm like, “Getting closer to my original disease-free self.”
[CL1-7] Release from my diseased self	CL 1 “Negative influence” and CL 2-7 “Approaching original self without disease” are difficult to name because the positives and negatives come together, but the negatives, as I mentioned, are very small in the whole. Rather, I would like to use the word “Release from my diseased self” as a word to sum up the whole thing, because it is only a derivative thing associated with coming through with a strong desire to be free from disease.

[Narrative of supplementary questions and survey feedback].

<Thank you very much. Let me ask you some more supplementary questions. You said “The first thing I gave up in my life was the piano.” Were there other things you gave up besides the piano?> I've had a lot to give up in my life. Sometimes I couldn't raise my hand even if I understood because I couldn't write on the blackboard at school, and sometimes I couldn't join club activities because I was worried about people's eyes. <Was there anything else?> When I grew up, I wanted to go on a working holiday, but when I looked at the requirements, it said that “healthy person,” and I wondered “how healthy is healthy,” so at last I couldn't apply. <So, you gave up your application?> Yes. And it's not that I don't think about marriage, but it puts the brakes on my head. My mother also has the same disease, which is hereditary, I thought that I could not involve anyone and that it would be better to end my life without having children, so from the time I was in junior high school, I thought it would be better not to get married. <Since junior high school...have you always felt the same way?> Now, I sometimes think that when I reach the age when I can't have a baby as a woman, I might rather be able to marry without restrictions. <Have you noticed any change in your feelings?> This is complicated. Living with this disease is hard and stressful because I can't do what I want to do. My older sister has the same disease, but she is, if I may say, an honest person, and when she felt unpleasant at school, she would go home and complain to mother in tears. And when I see my mother having trouble with it, I can't talk about my own. <Even if your family members have the same disease, you wouldn't talk about it, would you?> We talk about things like, “This kind of stretching sounds good,” but it was taboo to talk about the disease at length. The mother never expressed any weakness or says anything about her disease. I've come to think that I have no choice but to endure. <Both your mother and you have had to endure…> Yes, I think so. <I'm sorry to hear that...>.

<Finally, could you tell me about your thoughts on this interview?> Even if I told someone the name of the disease, no one would recognize it, and up until college, I couldn't tell my friends about the disease at all. Until my mid-twenties, when I tried to speak, I would sometimes cry, and when I spoke to someone, I couldn't speak at all because I felt like I was handing over something heavy that I had. This is the first time that I've talked like this. It was refreshing to talk about so many things. I'm not a good storyteller, so I'm surprised that I can talk this much.

The following is a Cluster-by-Cluster Interpretation of the results in Table 2.

##### 3.2.4.1 CL1 Interpretation

Of the 27 association items, Participant G classified two items labeled with negative images into CL1. Hence, this cluster was named “Negative Influence.” However, regarding time and financial burdens, the participants felt it is “drowned out compared to the benefits.” As such, we presume that the participant considers these items as self-investment to achieve her targets.

##### 3.2.4.2 CL2 Interpretation

Participant G's motivation for visiting Robocare Center was a sense of anxiety that she might not be able to continue her job, which involves a lot of standing work. The association item that was ranked to be most important was “No longer feeling anxious,” and the second was “Not having to quit my job.” She stated that her job is not only the foundation of her life but also a reason to live, her identity, and something that keeps her going. It is significant that she was able to avoid the loss of such vital things and that her sense of anxiety was eliminated through the use of HAL, and we anticipate that the event could have a significant impact on Participant G's future life.

##### 3.2.4.3 CL3 Interpretation

Participant G's first experience of “giving up because of disease” was when she had to give up playing the piano, which she had loved in her childhood. After almost 30 years of distancing herself from the piano, her turning point may have been “becoming easier to open my fingers” and “the change in my mind that I don't have to play like others.” Her words, “I loved it after all,” conveyed her great joy at being able to resume playing the piano.

##### 3.2.4.4 CL4 Interpretation

She described in detail how she had been dominated by vague anxiety because she was suffering from an intractable disease. She compared this to how she felt her body temperature. She gradually freed herself from being preoccupied with her disease to a state of mind in which she wanted to do something positively and actively. As she said in her self-insight, “It may be due to my age,” we analyzed that as she has gotten older, she has become better at dealing with disease and has become more flexible in her thinking.

##### 3.2.4.5 CL5 Interpretation

CL5 is the largest cluster with nine association items. She reported heartfelt reports of improvements in gait, trunk, and balance and that she started to enjoy walking more as she improved. We also interpreted it as an endorsement of not having to quit her job from the physical perspective.

##### 3.2.4.6 CL6 Interpretation

Participant G mentioned improvements related to feet and shoes and changes in life, such as going to the gym and using the stairs. Her statement that she was “naturally very active personality, but the disease has restricted me and balanced me” could be interpreted as a positive sense of her life living with the disease, which she shared with laughter. We felt that it was the new frontier of Participant G that she had reached after years of struggle.

##### 3.2.4.7 CL7 Interpretation

We interpreted this as an episode of further adaptation to the world surrounding her.

##### 3.2.4.8 Interpretation on the process of linking CL

Participate G mentioned that she has the mentality of her “original self” and “self who has become restricted and withdrawn after being diagnosed with the disease.” But in the end, the whole story was summarized as “Release from my diseased self.” These words may be a voice of joy that Participant G has steadily achieved her primary goal of improving her physical condition after years of suffering, or they may be words of prayer that express her strong desire for an ideal condition even though she knows that the disease is incurable.

### 3.3 Results of the survey for nine participants

Each of the nine participants' interviews lasted ranging from 3.5 to 5.5 h for the two sessions combined. No adverse physical or psychological effects were observed. Table 3, “List of Association Items of Nine Participants,” shows the results categorized by the three aspects (physical, mental, and social). Seven participants reported the most on the “physical” aspect, while two reported the most on the “mental” aspect. None of the participants wrote down the most numbers of “social” aspects. Table 4, “Classification of Association Items by three aspects,” classifies the association items into three aspects. We organized 67 items as “physical,” 56 items as “mental,” and 40 items as “social.” The following three conducted the classification: the researcher and two expert staff members (physical therapist, certified health exercise instructor) of the Tsukuba Robocare Center.

**Table 3:** List of association items of nine participants.

**Research participants**	**Physical aspects**	**Mental aspects**	**Social aspects**
**A**	• Legs are getting lighter and lighter • Legs are not getting caught on the ground anymore • Legs go up smoothly and can go forward • I fall less	• It's getting fun to walk • I want to keep going and get better • It gives me confidence that I'm working hard and rehabbing • Be a source of encouragement to oneself • The goal was revived again • I'm starting to wish I could let go of my walker someday • I became adventurous in trying to take a path I had previously avoided • When I get to this point, I can't give up until I let go of my walker • I thought this was enough because I was disabled, but now I want to work harder • I want my parents to know correctly that I am better than before	• There are more things I can do standing up that I used to do sitting in the house • I fell because I thought I could do something I couldn't do • At work, I've become more willing to express my opinions
**B**	• Now I can stand with HAL • My left leg is up now • My left leg can now bend (cross) in bed • My left hand can shake hands now • I was able to walk through rehabilitation • Rehabilitation helped me run • Now I can stand without HAL • I can sit by myself now • When I straightened my right side, my left side grew taut • The arch of my left foot started hurting	• Feelings became positive • There is hope of recovery • I was overjoyed • I was lucky to use HAL so early • I was lucky the doctor knew about HAL • My desire to move has changed to a conviction that I can move • I was surprised to see my body move	• I was relieved to be able to go to the toilet • I can take a bath by myself • I started going out actively. • The movement of housework (cleaning and cooking) became possible through rehabilitation • I moved to another hospital for rehabilitation
**C**	• I'm trying to raise my toes, but it's unfortunate that I still can't • I want to try training to build muscle • No more broken bones and sprains	• I was happy that my left leg moved with HAL. I was so moved • I'm getting more confident in my left foot • Meeting HAL makes me motivated • It's been a while since the onset of the disease, so I hope I get better little by little • I was impressed that I could walk without holding my left leg • I'm glad my physical function is back • When I was admitted to the hospital, I could walk on a walker, thank HAL • I was thrilled to be able to pedal a bicycle for the first time • There is not much change in the mental aspect because of the original spirit of challenge • I used to be a tough person	• I am now able to walk in socks • I used to have a lot of hobbies, and there have been no major changes in my life
**D**	• I was able to graduate from the wheelchair • It became possible to walk on my own • Now I can go up and down the escalator • I could walk from the Palace Hotel to Tokyo Station • The ability to walk with a cane broadened my range of motion • I got to take the elevator • Even with HAL, my legs are starting to rise • My right knee joint (paretic foot) became bent with HAL on • My head was tired while wearing HAL	• I was happy to graduate from the wheelchair • I have improved my ability to write and perform daily activities, and have a general feeling of OK • A sense of being OK because I am not a burden to society or my family • Being able to leave the bath lift made me feel less embarrassed • I'm so glad the language is almost back to normal • In the early days of my disease, I thought it would be difficult to walk on my own	• Public transportation is now available • No more trouble for my family, more pious • I was able to return to work • I can now drive a car
**E**	• I could let go of my cane • It became easier to walk • It was effective after training, but not the next day • Sometimes my body can't keep up with HAL's movements • Lately my arms have been shaking a lot	• I feel refreshed when I come here • The mental effect was greater than the physical recovery • It brightened me by coming • Coming through hoping not to get stuck	• Healthy and absent husband is nice. My family is liberated • I would like to come more (more times) if the usage fee becomes cheaper • I want a device that I can use for daily life
**F**	• The day after training, the tension in legs decreases slightly and becomes more relaxed • The pain goes away • Relieves body stiffness (flexibility • The device hit me and caused internal bleeding • Spasticity becomes stronger, but so does countervailing power, with a little better control • My body feels comfortable right after training • I feel a little pressure in my lower abdomen, which makes sitting easier • Able to reduce the loss of range of motion in the joint • Driving has become less difficult physically when coming to training	• Sometimes I'm disappointed by the difference between the image I have of myself and the reality • Going out has become less daunting • I feel uplifted • Fun to be in touch with cutting-edge technology • I am looking forward to the development of HAL as a medical device	• I enjoy eating out and shopping while coming to HAL • The purpose of going to HAL increased my activity • Increased conversation with others • I used HAL enthusiastically as part of my rehabilitation to go back to college • It was natural for me to do rehabilitation, and I chose HAL as one of the means • I can now plan my schedule including long travel times • I started to look more closely at my calendar • It costs money
**G**	• Fingers became easier to open • Stable and balanced body • My torso is getting firm • Achilles tendon lengthens so the back of the foot rests firmly on the ground • Less difference between the left and right sides of the body • Less sideways walking and more forward strength • Easier to stand, easier to walk • Less tiring • More strength in toes • It became easier to put my feet into shoes • Decreased legs deformities	• Easier to accept offers • Less time to think about disease • No longer feel anxious • I can think positively now • Able to remain fulfilling • Be able to keep the identity • Walking became fun • Feeling more flexible	• Increased financial burden • Less time for myself • Resumed playing the piano • Not having to quit my job • Easier to go out • Started going to the gym • I use stairs more often • Enjoy shopping before and after HAL
**H**	• The upper body was rather more trained than before the injury • The right part of my body is getting muscles • My torso is getting back to normal • My legs are getting stronger • The force of stepping on my right foot is getting stronger • The left leg from the hip joint down has become stronger • The left leg from the hip joint down has become stronger • MRI shows that the spinal column is fairly intact • Joints hurt when HAL is not tuned properly	• There is hope for recovery. Changed into conviction that I could recover • I feel more and more confident. • Looking at the HAL data led to confidence in functional recovery	• I'm starting to be able to press the accelerator on my car • Driving is getting easier • I started actively scheduling • I started handcycling. Schedule for Rounding Lake Yamanaka • I started oil massage and stretching
**I**	• It has become easier to put out the left foot and walk • I can go up stairs without a handrail • My body became more balanced • I was surprised to see my left big toe go up • I can check my walking by looking at HAL data • The training of the index finger of the left hand was not as effective as I expected • It is sometimes difficult to set up and fit HAL	• I learned to keep a never-give-up attitude. • I don't want to end up as the person who got injured, so I have hope to snowboard again • Being matter-of-fact because I accept myself as I am	• Coming to HAL has become a habit in itself, creating a rhythm of life • Coming to the shopping center for shopping, dining, etc. is also a pleasure for me

**Table 4:** Classification of association items by three aspects.

**Categories**		**Constructs**	**Specific examples of association items**
**Changes in physical aspects (67)**			
Functional improvements (60)	Improvement of body part (28)	Legs improvement (22)	Legs are getting lighter and lighter/My legs are getting stronger
		Torso improvement (4)	My torso is getting firm/The upper body was rather more trained than before the injury
		Hand Improvement (2)	Fingers became easier to open/My left hand can shake hands now
	Improvement of ability (21)	Improved walking ability (13)	It became easier to walk/It became possible to walk on my own/I fall less
		Improved balance ability (3)	Stable and balanced body/Less difference between the left and right sides of the body
		Improved mobility (3)	I can go up stairs without a handrail/Now I can go up and down the escalator
		Improved sitting ability (2)	I can sit by myself now/I feel a little pressure in my lower abdomen, which makes sitting easier
	Improvement of overall feeling (3)	My body feels comfortable (1)	My body feels comfortable right after training
		Less tiring (1)	Less tiring
		Relieves body stiffness (1)	Relieves body stiffness (flexibility)
	Others (8)	Other functional improvements (3)	MRI shows that the spinal column is fairly intact/The right part of my body is getting muscles
		Under Improvement (5)	It was effective after training, but not the next day
Others (7)	HAL setting matters (5)	Difficulty fitting HAL (3)	It is sometimes difficult to set up and fit HAL/The device hit me and caused internal bleeding
		Difficulty tuning HAL (2)	Sometimes my body can't keep up with HAL's movements
	Others (2)	Others (2)	I can check my walking by looking at HAL data/I want to try training to build muscle
**Changes in mental aspects (56)**			
With functional improvements (50)	Motivation (16)	I want to be better, I don't give up (7)	I want to keep going and get better/I learned to keep a never-give-up attitude
		Encouraging and uplifting (5)	Meeting HAL makes me motivated/Be a source of encouragement to myself/I feel uplifted
		Feeling positive (4)	I can think positively now/Feelings became positive/It brightened me by coming
	Confidence (8)	Confidence building (6)	It gives me confidence that I'm working hard and rehabbing/I feel more and more confident
		General feeling of OK (2)	A sense of being OK because I am not a burden to society or my family
	Hope (5)	Hope is rising (3)	There is hope of recovery/The goal was revived again
		Changed from desire to conviction (2)	My desire to move has changed to a conviction that I can move
	Others (21)	Happy and impressed (12)	I was happy that my left leg moved with HAL. I was so impressed
		Anxiety reduced (4)	No longer feel anxious/Less time to think about disease/Easier to accept offers
		Walking became fun (2)	It's getting fun to walk/Walking became fun
		Good luck meeting HAL (2)	I was lucky to use HAL so early/I was lucky the doctor knew about HAL
		Feeling more flexible (1)	Feeling more flexible
Original nature (3)	Original nature (2)	Self-acceptance (1)	Being matter-of-fact because I accept myself as I am
		Original nature (2)	There is not much change in the mental aspect because of the original spirit of challenge
Others (3)	Others (3)	Others (3)	Fun to be in touch with cutting-edge technology
**Changes in social aspects (40)**			
Improvements of ADL/QOL (26)	ADL improvement (17)	Improvement in activities of daily living (6)	I use stairs more often/I can take a bath by myself/I was relieved to be able to go to the toilet
		To be able to manage the schedule (4)	I can now plan my schedule including long travel times/I started actively scheduling
		It became easier to go out (4)	Public transportation is now available/I started going out actively/Easier to go out
		Driving a car (3)	I can now drive a car/Driving is getting easier
	QOL improvement (9)	Started hobbies (4)	Resumed playing the piano/Started going to the gym/I started handcycling
		Return to work and school (3)	I was able to return to work/I used HAL enthusiastically to go back to college
		More Conversations (2)	Increased conversation with others/At work, I've become more willing to express my opinions
Increased burden (4)	Increased burden (4)	Increased burden of money and time (4)	I want to come more often if the fee was lower/Increased financial burden/Less time for myself
Increased pleasure (3)	Pleasure of coming (3)	Pleasure of coming (3)	Enjoy shopping before and after HAL/I enjoy eating out and shopping while coming to HAL
Reducing the burden (2)	Reducing the burden on family (2)	Reducing the burden on family (2)	Husband is healthy and away from home is nice. My family is liberated
Others (3)	Others (3)	Others (3)	It was natural for me to do rehabilitation, and I chose HAL as one of the means

Narrative summaries for each of the eight participants except for G, are shown in Table 5.

**Table 5:** Narrative summaries of the eight participants (excluding G).

**A**	My legs have improved, and I can now do things standing up that I used to do while sitting at home. I also fall less often. Thanks to HAL, my condition is getting better, and I've started to enjoy walking. Since I've come this far, I don't want to give up until I no longer need a walker. A while ago, I thought I could walk better than I actually could, so I pushed myself too hard, ended up falling, and got slightly injured. However, I don't see this as a bad experience. In the past, I sometimes thought, “I have a disability, so this is good enough.” But recently, I've been trying harder at work and have started expressing my opinions more actively. Improvement isn't just physical—it's also mental. I believe that by improving in both aspects, I can set new goals for myself. Right now, I'm still in the process of improving and continuing to challenge myself to reach new milestones.
**B**	(B, started using HAL at the hospital where they were admitted, began visiting the Tsukuba Robocare Center after being discharged) After experiencing a second brain hemorrhage, the doctor told me that I would never walk again. The hospital did not have HAL, but my husband convinced the doctors very hard until the hospital introduced it and allowed me to use it. When I first stood up with HAL, I was excited. Many hospital staff gathered to watch, and some even hugged me. Later, I felt relieved when I could go to the bathroom by myself. When I finally stood up without HAL, my hope of “I want to move” turned into the confidence that “I can move.” On the 38th day of hospitalization, I was able to walk without a cane. Having access to HAL early on was an incredible stroke of luck. I would like to express deep gratitude to the professor who developed it. Now, I encourage others with the same condition who visit the Tsukuba Robocare Center. If given the opportunity, I would love to publish a book about my experiences.
**C**	I happened to learn about HAL on TV and called the next day to arrange a visit to the Tsukuba Robocare Center. Since I hate losing, I want to try everything I can. Having lived with polio for a long time, I hope to see gradual improvements. I was excited when my left leg moved with HAL, as it had never moved before. Recently, I no longer suffer from fractures or sprains. My core has become stronger, my balance has improved, and I no longer slip, so I can now walk even while wearing socks. My frostbite has also improved. I was overjoyed when I was able to pedal a wheelchair-type bicycle for the first time at the Tsukuba Robocare Center. Moving forward, I would like to try training with HAL to make walking even easier. I have always been determined and eager to take on challenges, so I don't feel much of a change mentally. Likewise, since I have always had many hobbies, there hasn't been much change in my daily life. However, I am grateful to have discovered HAL and am determined to continue making progress with it.
**D**	(D, used HAL at the hospital where they were admitted and continued training at Tsukuba Robocaare Center after being discharged) When I first used HAL Single-Joint at the hospital, I was surprised that the knee joint on my paralyzed side was able to bend so quickly. After being discharged, I continued training at Tsukuba Robocare Center. I was overjoyed when I no longer needed a wheelchair. At the time, I never imagined I would be able to walk without a cane, but as I gradually improved, I began to see the possibility of walking independently. While continuing to use HAL, I worked on improving my walking ability by using escalators and public transportation. As I became able to perform daily activities, I started to feel that I was “almost OK.” Being able to drive again was not only a change in my mobility but also a significant mental shift. When I first returned to work and still needed my family to drive me, I felt I was half a man. However, once I could drive myself, it felt like “an ogre armed with an iron club (Japanese idiom of making something powerful evenmore powerful)” Since aging is inevitable, I want to continue using HAL to maintain and improve my abilities.
**E**	After being discharged from the hospital, I initially relied on a cane, but since starting training with HAL, I have been able to walk without it and no longer need my long leg brace. However, I still use a cane when going out as a precaution. Even though I know I should exercise at home, I find it difficult to do so, which is why I come to Tsukuba Robocare Center. I also come here to refresh myself mentally. As the saying goes, “A good huband is one who's not sick and not around,” and while I'm here, my wife gets a break too—so it benefits both of us. I feel that the mental benefits have been even greater than the physical recovery. I've become more cheerful and positive. However, once I return home, I lose the effects of the training, so I wish there were a device I could wear in daily life. If the fees were lower, I would come here every day.
**F**	While in college, I was injured in a motorcycle accident and, during my leave of absence, I came to Tsukuba Robocare Center to train with HAL, driven by the desire to improve. I was able to return to college, but during that time, the distance made it impossible to continue with HAL. After graduating, I returned to my family home and resumed my HAL training. Due to my spinal cord injury, I have no strength below the solar plexus. As a result, the relief I feel when sitting after using HAL is significant. I also believe that HAL helps reduce the tension in my legs, alleviates pain, and lifts my spirits. I have not given up on the hope of eventually standing or walking, which is why I continue coming to the center. Since I started coming here, my social interactions have increased, and going out has become less of a hassle. I now find myself eating out and shopping on my way home. HAL has helped me establish a routine, allowing me to manage my schedule more effectively, both before and after my sessions. In the past, when my physical condition was worse, my daily life felt more random, and I would sometimes forget my HAL appointments. Now, that is no longer an issue. To aid in hand rehabilitation, I started playing para-table tennis 4 years ago, and I occasionally compete in international tournaments. More recently, I've also begun wheelchair tennis. As my body stabilizes, I'm excited to try even more activities. I believe there is a positive correlation between my physical recovery and my engagement with society. With the goal of finding a job in the future, I have begun job hunting.
**H**	The biggest change among these three points is the mental transformation I've experienced since starting HAL training. I believe it's common for people undergoing rehabilitation to have doubts and fears about whether they will really improve. However, because HAL visualizes the data, it gives me hope that improvement is possible. After using it a few times, I gained confidence, believing that I would recover and be able to regain my abilities. This is why I don't give up. I consider this to be the greatest progress I've made since coming here. Initially, my nerves were in a state of confusion, but as they calmed down, the numbing pain gradually faded. On the right side, I believe the nerves are being sorted out and becoming more stable. The left side still needs more time, but my hip joint has become more solid. I now feel that, as long as the signals are being sent, it's just a matter of time before they will be properly organized, and it will depend on what triggers that change. After leaving the hospital, I was initially reluctant to interact with others or go outside because I was worried I might inconvenience people. However, over the past 6 months, I've been able to move my legs much better, and I can now sit anywhere. When I go out to eat, I no longer want to eat in my wheelchair, so I've changed my behavior to sitting at a restaurant table and eating at eye level with other people. Recently, I've become more proactive in making plans. I started handcycling and am planning to cycle around Lake Yamanaka. I feel like my body and mind are finally in harmony.
**I**	Since starting HAL, the physical changes have been significant. First, I was surprised to see that my left big toe could lift. After that, it became easier to move my left foot, walking became easier, and it became easier to maintain my body's balance. I can now go from the first floor to the second floor without a handrail, even though I wobble. I think it's helpful to be able to check my walking while looking at the data on the HAL monitor. Coming here has become a habit and has established a rhythm in my daily life. The fact that the center is located in a large shopping mall makes it even more enjoyable. Window shopping has turned out to be excellent rehabilitation. Since I have to make do with my body as it is now, I don't feel discouraged, nor do I have unrealistic expectations. I just accept things as they are and take a calm, measured approach. I sustained an injury while snowboarding, so I'd like to visit the snowy mountains once more. I don't want to be defeated by my injury, or rather, I don't want things to end this way. If I put it in a more positive light, I think I've learned to keep the spirit of not giving up. Until my children become adults and start to earn their own income, I hope to continue working while gradually improving with the help of HAL. Since there were no other rehabilitation options, I began coming here with the hope of getting better. I can say that I accept myself as I am and stay calm, but perhaps that is because I can see something ahead of me and that thing seem hopeful.

## 4 Discussion

### 4.1 Discussion of the survey results for nine participants

Since “Neuro HALFIT” is a program to support functional improvement, HAL users use the service primarily to improve or enhance their physical aspects. All nine individuals, including the seven with disability certificates, as shown in Table 1, reported “improvements in body parts,” such as legs, hands, and trunk, and “improvements in physical abilities,” such as walking, mobility, balance, and sitting. Some also reported improvements in their “overall physical feeling,” such as “feeling more comfortable,” “better endurance,” and “less stiffness.”

Participants also showed improvement in their mental health. The content (of functional improvement) varied from “I am happy/impressed,” “I want to get better,” “I feel more positive,” and “Walking has become more enjoyable.” There were also reports of a solid response to the recovery: “My desire to move has changed to my conviction that I can move (B)” and “There is hope for recovery. Changed into a conviction that I could recover (H).”

In addition, some participants listed more mental improvement than physical improvements in their association item (A, C) or written association item that “The mental effect was greater than the physical recovery (E).” These cases have one thing in common: The participants who felt more mental improvement tended to continue using HAL for a long time. Once they achieve a certain degree of improvement, they work to maintain their physical condition, which seems to be the time they experience mental enrichment (C: “Meeting HAL makes me motivated”/E: “I feel refreshed when I come here”).

In terms of social aspects, various changes (positive changes) can be seen in their lives, such as improvement in ADL (activities of daily living, housework, going out, driving a car) and improvement in QOL (hobbies, return to work, return to school, conversation). To give some examples from reports in this aspect, “(because my trunk has become stronger, my balance has improved, and I have stopped slipping),” I am now able to walk in socks (C), “Resumed playing the piano (G),” and “I started hand-cycling (H).” In addition, there are also cases that led to significant life events, such as returning to school and graduating while using HAL after becoming a wheelchair user due to an accident when the participant was in college (F) and being able to return to work (D).

Reports such as “Using HAL has become a habit in itself, creating a rhythm of life (I)” and “I started to look more closely at my calendar (F)” were common narratives of people who came to the center, as they tend to come in on fixed days and times, which became the center of their entire lives. Others reported that their recovery had helped reduce the burden on their families. Such as, “No more trouble for my family, more pious (D)” and “Healthy and absent husband is nice. My family is liberated (E).”

Tsukuba Robocare Center is located in a large shopping center, and some participants (F, G, I) reported that they enjoy shopping and eating before or after using HAL, which is one of the motivations for continuing “Neuro HALFIT.”

On the other hand, some pointed out that the price to use HAL is high (F, G) and want to come more often if “Neuro HALFIT” is cheaper (E).

### 4.2 Discussion of participant G's narrative

Tracing Participant G's narrative with the keywords “give up/don't give up” reveals the following narrative. “Struggling with disease and loneliness, I lived on while giving up many things. I had given up on curing the disease as there was no cure. My body has gradually improved in the 3 years since I started HAL. I did not have to give up my job that I was about to give up. I can once again play the piano that I once gave up. I also gave up marriage, but I am starting to think it is possible as I get more mature according to my age.” We felt that she is now at a turning point where she is changing from “a life of giving up” to “a life of trying as hard as she can without giving up.” We analyzed that these turning points were mainly brought about by “improvement and enhancement in physical, mental, and social aspects” and “becoming more mature as a person.” We will discuss these two points further.

Participant G's disease is a rare hereditary neurological disease, and modern medicine has not found a cure or treatment to slow its progression. However, functional improvement with HAL (Lower Limb Type/Single Joint Type) has resulted in improvement of the legs and hands themselves, stability of the trunk, and physical improvements such as reduced lateral sway, easier walking, and less fatigue. The results showed a wide range of mental changes, from “no longer feel anxious, started to enjoy walking” to “becoming more accepting, more positive, more flexible” to being able to keep “reason to live, identity and the energy keep on going.” Then there were social changes such as “I didn't have to quit my job, I started playing the piano again, I started going to the gym, I started to use stairs, it became easier to go out.”

In Participant G's narrative, there were several places where she plainly stated that mental and social changes (including behavioral and lifestyle changes) occurred after the physical improvement. The following are those extracted comments.

- I had difficulty going outside when moving was difficult, but now I am more outgoing and can think more positively (physical → mental).
- Now that I am better after using HAL, It's almost like I am coming down a little from the peak of my fever. As mentioned in the association item, my feelings have become more flexible. It may be due to my age, but I don't need perfection (physical → mental). It has become easier to accept invitations from friends (physical → mental → social).
- The condition of my legs is improving, and I feel like I should walk instead of drive (physical → mental → social).
- I am starting to enjoy walking and feel a change in walking (physical → mental → social).
- I feel more positive and want to try something, so I started playing the piano again (physical → mental → social).
- My feet were deformed, and there was a difference between my left and right foot, so I had difficulty wearing shoes. The deformity is starting to be removed, and wearing shoes is now easier. Moving and doing something became easier, so I started going to the gym (physical → social).

The comments above suggest that the improvement of the physical aspect triggered a virtuous cycle of “improvement and enhancement” in which the three aspects are interconnected and influence each other, spiraling up to the next step.

In addition, there were changes in her mind, such as “I don't have to play the piano like others,” “I don't have to be perfect,” and “the disease has restricted me to make me balanced,” suggesting that there has been a positive sense of living with the disease while accepting it. These mental changes may be a new frontier brought about by her maturity as a person, as she has become better able to deal with her disease as she has gotten older, and with that, she has acquired a new sense of values to reassess her life in a positive and affirmative sense.

The findings above suggest that the alignment of both “improvement and enhancement in physical, mental, and social aspects” and “maturity as a person” has brought about a change from a “goal-oriented” mentality that aims for an ideal goal to a “process-oriented” mentality.

The narrative, in line with the dendrogram and those about what she had given up in her life, drawn out by questions, vividly represented a part of Participant G's journey so far and revealed the painful feelings that she had not been able to talk about due to her hereditary and rare disease. The interview facilitated a natural form of self-disclosure, as Participant G commented, “This is the first time I have talked about something like this,” and “I'm surprised myself.”

### 4.3 Discussion on “narratives in which the physical, mental, and social aspects are interrelated”

In Participant G's case, we saw a positive “improvement and enhancement cycle,” which was triggered by physical improvement followed by mental and social improvement. In this cycle, the three aspects are interrelated and affect each other to form an upward spiral toward the next step. Similarly to the previous Section (4.2), when examining the other eight participants, we observed a similar trend in the narratives of the other eight participants. We listed the excerpts of this trend in Table 6, “Excerpts of narratives in which the physical, mental, and social aspects are interrelated (8 cases).” These narratives suggest that mental and social changes (including behavioral and life changes) have occurred because of physical improvements caused by HAL.

**Table 6:** Excerpts of narratives in which the physical, mental, and social aspects are interrelated (8 cases/excluding G).

**Research participants**	**Excerpts from 8 narratives**	**P (physical), M (mental), S (social) relationships**
A	• It's getting better and better, so even the roads I thought were hard to walk feel like an adventure. I became adventurous. • Before HAL, it was just rehabilitation or a little exercise to maintain my strength, but after I got better with HAL, I started to enjoy walking. • Improvement can be physical or mental. With both of these improvements, I think I can set a new goal.	• P → M • P → M • P · M → M
B	• I was very pleased when I was able to walk with HAL on. “Good!” right? • I was wearing a diaper, but I was able to stand on my own and go to the toilet, so I was relieved. I appreciate it. • When I first stood using HAL, I felt positive. Since I had been in bed for more than 2 weeks, my eyes just went up and I was like, “Oh?!” • I thought it was possible to move with a robot. It was healed in a month, so I didn't feel like suffering.	• P → M • P → S → M • P → M • P → M
C	• I was very impressed when my left foot moved for the first time, which had not moved at all. My left foot has somehow become more confident. I was anxious because I had a feeling of what I would do if I broke out. That's why I've fallen before. I'm glad I don't have that now. • Since about last year, I've been able to walk in socks. I've been barefoot all year. I felt like I was slipping, and I was afraid to fall, so I couldn't wear them. As my left leg grew stronger, I stopped slipping and started to think I was OK. I've had chilblains all year, but now that I can wear socks, it's warmer.	• P → M • P → M · S
D	• When it comes to being able to do most things around me, such as writing or simple daily activities, I am able to do most things. It means that I am generally OK. I feel that I am not a burden to society or my family. • Being able to ride in a car was a behavioral change, but it was also a mental change. It's a limitation of physical ability, and after a disease, I feel like there's nothing I can do like a normal person. But if I drive, I can go anywhere with no handicap. The best part was that I didn't have to rely on my family to go to work. When I say return to work, it's odd to bother my family on the way back and forth, and if I can't do this, there are distortions in many ways. By being dependable. It's not a magic wand, but it's a “As long as you have this, the ogre will have a gold bar.” Using the vehicle, a tool of civilization, I was almost as capable as a healthy person. It's an extremely unreliable ogre, but if you add a gold bar called an automobile to it, is it completely normal?	• P → M • P → S · M
E	• I think it's more a mental benefit than a physical recovery. I can be positive because of this. If I'm good physically, I'm good mentally.	• P → M
F	• My body has improved and I can now plan my schedule, including long travel times. I've been able to manage my normal schedule, like getting ready for this time to be here, being able to put in this errand. I manage it with a calendar app on my smartphone, but now I often look at my calendar too. Until now, I've been living on a hit-or-miss basis. • I got to go out because I trained at HAL and became active. My range of activities expanded and my involvement with society expanded. • Now that my body is more stable, I can go a little farther, or I can do these things, so I'll try what I've developed. • It's like not only one (physical) but both (physical and social) together, it's like this is what happens (I start to want to do a lot of things). Like, they're each other's foundation. Does it mean that there is a positive correlation between being physically better and social interaction? • It means that the rhythm of life and physical changes are linked.	• P → S • P → S • P → M • P → S/S → P • P → S/S → P
H	• The MRI shows that the spinal column is pretty much back to normal, and it's pretty clear. From the side, when the nerve muscles were injured, there was blackness along the way and they became thinner, but the thickness has returned to about full recovery. That made me more and more confident. • After I left the hospital, I was too unwilling to deal with people or go out. I was so indebted to them for being unable to move that even if I were to go out for a drink or play with my friends, I would cause them trouble. I used to go to the toilet with their arms around me, but in the last 6 months I've been able to move my legs a lot, and I don't need their help. I wasn't sure if it wasn't a good location, but now I can sit anywhere. I don't want to eat in a wheelchair when I go out to eat, so my behavior has changed to sitting in a chair and eating from the same angle as my friend if the chair in the restaurant is soft. Of course, there are changes in my physical strength and functions, but the biggest change is in my mind, and I think that's the most important thing. • It's been over a year and a half since I was injured. When I asked my doctor how long I could recover after surgery, he said, “There's about a 5% chance.” This may be comforting though. That's what I was told when I heard that people with spinal cord injuries are rarely able to stand up. You think about it in your own way, and it may be your personality, but you've never been toned down. From the time I was in the hospital, I was determined to get well. I've never been too down, partly because I'm optimistic that something will be done. Having said that, after a year and a half, I still had anxiety about when it would be. Since I met HAL this time, I've been thinking more positively. The range of action has broadened. I was originally confident, but I became more and more confident. Even if we do the same thing, I think the results will be different if we do it with confidence and not.	• P → M • P → M · S • P → M · S
I	• To put it in a positive way, does that mean I never give up on anything? In my head, I think I can do it. I think it means that my physical condition is getting better and I can continue to have a feeling of never giving up. I can say that I am matter-of-fact because I accept myself for who I am, but I think I can be matter-of-fact because I see something beyond that. I think it's because I have hope in this.	• P → M

Narratives such as “Improvement can be physical or mental. I think both improvements lead to the creation of a new goal (A)” “If my body improves, my mind improves (E)” and “Not just one (physical aspect) but both (physical aspect and social aspect) come together to be like this (I want to do various things). Each of them will become a foundation for the other. I suppose it means a positive correlation between physical improvement and social involvement (F),” which tells us plainly that physical, mental, and social aspects will improve and enhance each other rather than develop independently.

Narratives like “I think the mental effect is greater than the physical recovery (E)” and “Of course, there are changes in my physical strength and functions, but I think the biggest change is in my mind, and that's the most important thing (H)” are notable for emphasizing the importance of mental over physical changes.

### 4.4 “Mutual feedback structural model in physical, mental, and social aspects”

**Figure 4:**
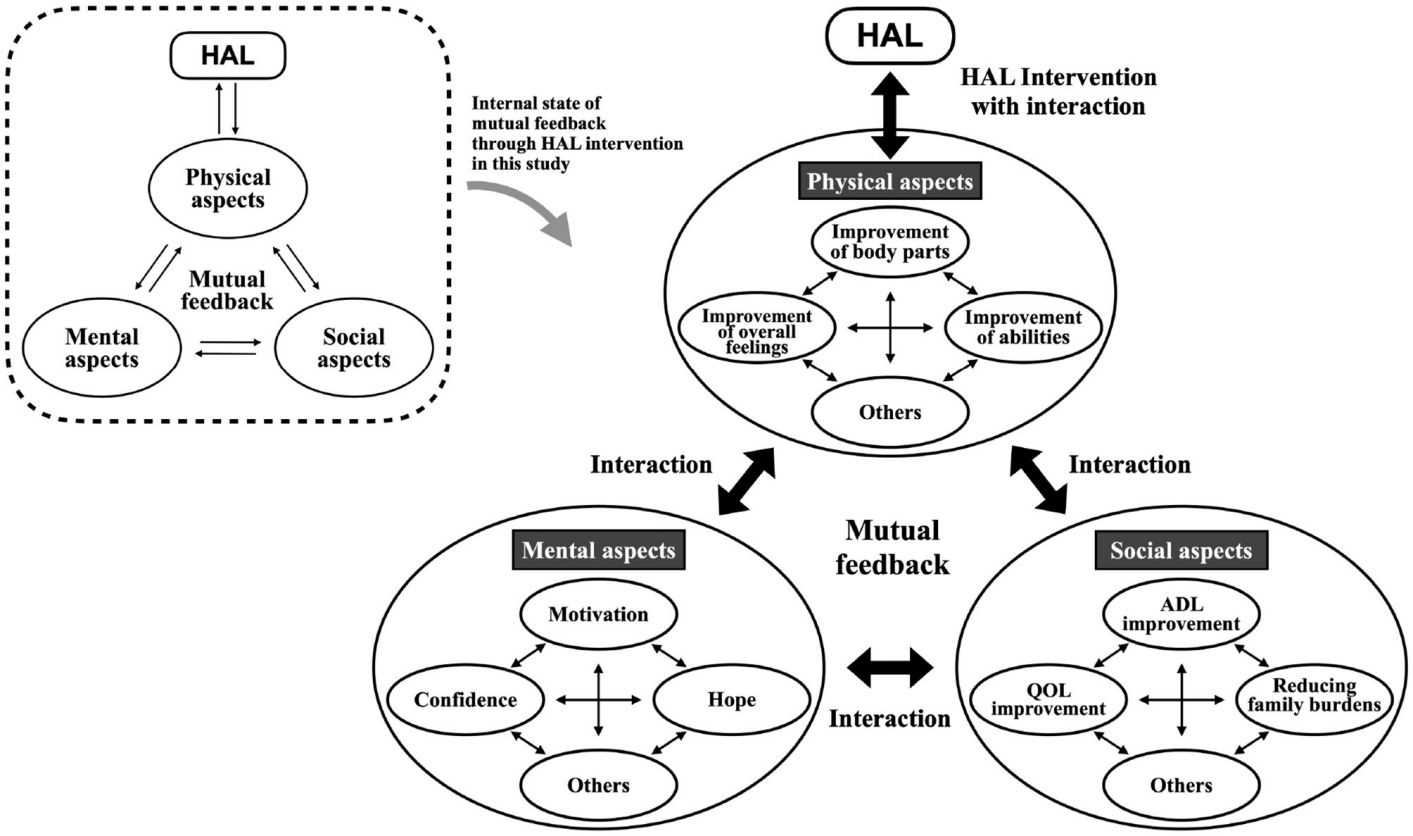
Mutual feedback structure model in physical, mental, and social aspects through HAL intervention.

The positive cycle of “improvement and enhancement,” in which the physical, mental, and social aspects are interconnected and influence each other and form an upward spiral toward the next step, was seen in everyone's narratives, including Participant G. This is shown in a conceptual diagram as a “Mutual feedback structural model in physical, mental, and social aspects” using keywords in Table 4 (Figure 4).

HAL users are considered to have achieved the primary purpose of using the service to improve their physical condition to some extent and continue to improve their physical condition. Improvement in physical condition led to mental progress, such as gaining motivation, hope, and self-confidence. In addition, ADL and QOL were improved, and it was shown that life improved and positive behavioral changes occurred. These three aspects are thought to influence each other and circulate mutually, leading to the next step toward improvement and enhancement.

The mutual feedback structure model revealed in this study suggests the universal possibility of cyclical continuous improvement of physical, mental, and social function through the utilization of technology such as HAL that breaks through the commonly accepted limit of improvement from standard-of-care treatment and other means of care. The interactions between the physical improvements and mental and social changes form a virtuous cycle that feeds into itself, promoting even further positive change that is vital to these patients.

## 5 Conclusion

The narratives of the participants, which were analyzed and visualized through the interview survey, are not intended to measure the HAL users' impressions at a certain point in time or changes before and after the use of HAL but rather to provide valuable data for understanding how they have changed over time using HAL. It became clear that “Neuro HALFIT,” which was initially intended to improve physical function, also brought about mental and social improvements.

It is challenging to determine the extent to which patients who have been disabled due to accidents or diseases can return to their original physical condition. Many who come to “Neuro HALFIT” have completed regular hospital rehabilitation. They also tend to be those who doctors told that they could not be expected to recover further, diagnosed with neuromuscular diseases for which there is no treatment, or use wheelchairs due to difficulty in walking. The nine participants in this study all fell into one of these categories. It is vital for such people to participate in society in a way that fits their needs by turning the process of the “Mutual feedback structure model” without losing hope while enjoying their daily lives and feeling a sense of purpose. The most critical significance of using HAL is to support this process. Although the narratives of the nine participants are diverse, they all share this point in common.

In this study, during the interview survey, we also visualized the narratives using mathematical engineering methods (cluster analysis, dendrogram) based on the similarity distance matrix between the association items, and elicited them by deepening the narratives through counseling methods and captured the state of change in the physical, mental, and social aspects of the subjects. This proposed a new method, but at the same time, it has the limitation that it requires the level of counseling at the level of a clinical psychologist and that it takes a long time.

Another limitation of this study is that it cannot be said to reflect the reality of all HAL users because it only included those who continued with HAL and did not include those who improved their function with HAL and completed it, or those who discontinued HAL because it was not a good fit, thus causing a selection bias.

## Data Availability

The original contributions presented in the study are included in the article/supplementary material, further inquiries can be directed to the corresponding author.
